# Selenium-enriched *Bifidobacterium longum* DD98 effectively ameliorates dextran sulfate sodium-induced ulcerative colitis in mice

**DOI:** 10.3389/fmicb.2022.955112

**Published:** 2022-08-05

**Authors:** Yongjia Hu, Xueli Jin, Fei Gao, Ting Lin, Hui Zhu, Xiao Hou, Yu Yin, Shidong Kan, Daijie Chen

**Affiliations:** ^1^School of Pharmacy, Shanghai Jiao Tong University, Shanghai, China; ^2^State Key Laboratory of Microbial Metabolism, Shanghai Jiao Tong University, Shanghai, China

**Keywords:** selenium-enriched *Bifidobacterium longum*, DD98, probiotic, IBD, DSS-induced colitis, gut microbiota

## Abstract

The pathogenesis of ulcerative colitis (UC) is complicated with impaired intestinal epithelial barrier and imbalanced gut microbiota. Both selenium and probiotics have shown effects in regulating intestinal flora and ameliorating UC. The objective of this study is to investigate the alleviating effects of Selenium-enriched *Bifidobacterium longum* DD98 (Se-*B. longum* DD98) on dextran sulfate sodium (DSS)-induced colitis in mice and explore the underlying mechanism. After treatment of *B. longum* DD98, Se-*B. longum* DD98, and sulfasalazine for 3 weeks, the disease severity of UC mice was decreased, with colon lengthened and pathological phenotype improved. The expression of pro-inflammatory cytokines and oxidative stress parameters were also decreased. Thus, Se-*B. longum* DD98 showed a stronger effect on relieving the aforementioned symptoms caused by DSS-induced colitis. Exploration of the potential mechanism demonstrated that Se-*B. longum* DD98 showed higher activities to suppress the inflammatory response by inhibiting the activation of the toll-like receptor 4 (TLR4), compared to *B. longum* DD98 and sulfasalazine. Se-*B. longum* DD98 also significantly improved the intestinal barrier integrity by increasing the expression of tight junction proteins including ZO-1 and occludin. 16S rDNA sequencing analyses showed that Se-*B. longum* DD98 improved the diversity of the intestinal flora and promoted the abundance of health-benefiting taxa including *Lachnospiraceae*, *Lactobacillaceae*, and *Prevotellaceae* in family level. In conclusion, compared to *B. longum* DD98 and sulfasalazine, Se-*B. longum* DD98 showed stronger therapeutic effects on DSS-induced colitis in mice and might be a promising candidate for the treatment of UC.

## Introduction

Inflammatory bowel disease (IBD), comprising Crohn’s disease (CD) and ulcerative colitis (UC), is characterized as a chronic and idiopathic inflammatory disease affecting the ileum, rectum, and colon (Ungaro et al., 2017). The pathogenesis of UC is not well-understood and usually complicated by multi-factors including genetic predisposition, intestinal flora dysbiosis, epithelial barrier defects, immune susceptibility, and environmental elements (Hindryckx et al., 2016). It is reported that the prevalence of IBD is increasing worldwide, posing a significant challenge to medical industries (Molodecky et al., 2012; Kaplan and Ng, 2017). Furthermore, as an incurable intestinal inflammation, IBD contributes to colorectal cancer development through a dysregulated form of tissue repair or aberrant activation of inflammatory pathways (Elinav et al., 2013). The application of therapeutic drugs for UC patients, such as aminosalicylic acids, corticosteroids, immunosuppressants, and biological agents, was limited for their poor efficacy and potential adverse drug reactions (Meier and Sturm, 2011).

In recent years, the correlation between compositional and metabolic changes in the gut microbiota and UC has been investigated. Multiple studies reported that the diversity of intestinal microbiota and the proportional abundance of *Firmicutes* to *Bacteroidetes* or *Proteobacteria* was decreased in UC patients (Sartor and Wu, 2017). Besides, the metabolites of gut microbiota such as short-chain fatty acids (SCFAs), bile acids, and tryptophan catabolites, which exert a protective function in maintaining the gut barrier integrity and immunity homeostasis, are significantly reduced during the development of UC (Ni et al., 2017; Agus et al., 2018). Probiotics refer to the live microorganisms that confer a health benefit on the host if taken in a sufficient amount (Hill et al., 2014). Nowadays, the application of probiotics such as *Bifidobacterium* were confirmed to exert an important role in UC (Sartor, 2004). For instance, probiotics increased intestinal biodiversity and restored gut microbiota dysbiosis in colitis mice (Bian et al., 2019). Administration of *Bifidobacterium longum* inhibited inflammatory pathway activation in lamina propria cells and downregulated inflammatory mediators expression (Bai et al., 2006). In addition, probiotics alleviated symptoms of UC in DSS-colitis rats by improving gut barrier integrity and increasing SCFAs content (Sanders et al., 2019; Li et al., 2022).

Selenium (Se) is an essential trace element for human health. Selenoproteins, the main active form of selenium in the human body, are famous for their anti-inflammatory properties and immunomodulatory effects. Therefore, Selenoproteins were used in the prevention and treatment of autoimmune, allergic, cardiovascular, and chronic inflammatory diseases (Huang et al., 2012; Kuria et al., 2022). It is demonstrated that the patients with IBD were commonly found with selenium deficiency (Han et al., 2017). Besides, the efficacy of selenium supplement on UC patients and DSS-induced colitis animal models has been proven by many studies (Zhai et al., 2018a; Khazdouz et al., 2020). Krehl et al. (2012) reported that selenium mitigated the severity of DSS colitis by enhancing GPX2 activity. Zhu et al. (2016) demonstrated that selenium-containing phycocyanin significantly decreased the level of inflammatory cytokines and oxidative stress. Furthermore, selenium also regulated the composition of gut microbiota, thus restoring the flora dysbiosis in colitis (Zhai et al., 2018a).

Taken together, previous studies have demonstrated that both probiotics and selenium have improvement effects on UC. Se-*B. longum* DD98, a selenium-enriched probiotic, obtained through applying Na_2_SeO_3_ into the process of *B. longum* DD98 fermentation, thus converting inorganic selenium to organic selenium. Our previous studies showed the efficacy of Se-*B. longum* DD98 in antibiotic-induced intestinal dysbacteriosis and type 2 diabetes (Zhu et al., 2019; Zhao et al., 2020b). In this study, we investigated the effect of Se-*B. longum* DD98 on the prevention and treatment in DSS-induced colitis mice. Because of the situation that the use of selenium is limited by its potential toxicity in excessive intake and wide employment of probiotics in UC treatment, we compared its effects with pure *B. longum* and sulfasalazine (one of the first-line aminosalicylic acid drugs) to find out whether the Se-*B. longum* DD98 would show superior functions in relieving DSS colitis. Furthermore, mechanisms related to the level of inflammatory response and oxidative stress, the expression levels of tight junction proteins, and gut microbiota composition were also evaluated to explore the prevention and therapeutic effects of Se-*B. longum* DD98 in DSS-induced colitis mice.

## Materials and methods

### Preparation of selenium-enriched *Bifidobacterium longum* DD98

Selenium-enriched *Bifidobacterium longum* DD98 was prepared in our laboratory. The origin of the DD98 strain and preparation procedure were described in our previous study (Zhu et al., 2019). In brief, a high-throughput screening approach was used to select the DD98 strain. Se-*B. longum* DD98 was enriched by applying DD98 strain in the 24 h fermentation of culture medium containing 8.5 μgmL^–1^ Se (as Na_2_SeO_3_). At the end of fermentation, the bacterial cells were harvested, washed, and then freeze-dried for subsequent administration. The viable count of bacteria in the fermentation broth was determined by colony counting in reinforced clostridial medium (RCM) agar plates. The total selenium content is determined by the iCAP-Q inductively coupled plasma mass spectrometer (ICP-MS, Thermo Fisher Scientific, MA, United States). In this study, the total Se concentration and CFU of Se-*B. longum* DD98 were 0.38 mg/g and 2 × 10^9^ CFU/g, respectively.

### Animal experiments

**FIGURE 1:**
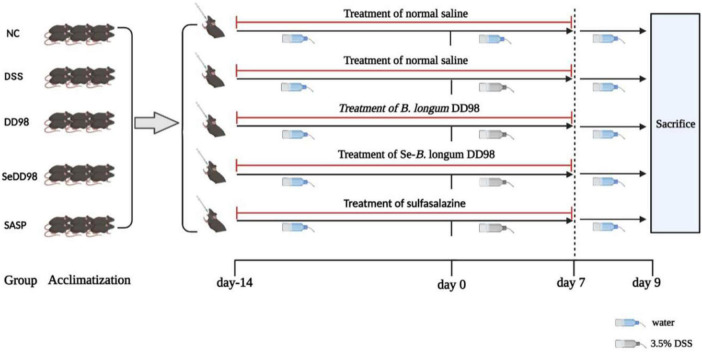
Scheme of the animal experimental design.

The animal experimental design is summarized in Figure 1. All animal experiments were performed by confirming to the relevant regulations and permitted by the Institutional Animal Care and Use Committee (IACUC) of Shanghai Jiao Tong University (NO. A2021046). Thirty male C57BL/6 mice (6–8 weeks, 20–22 g) were purchased from Beijing Vital River Laboratory Animal Technology Co., Ltd. (Beijing, China). All mice were acclimatized at 25 ± 2°C in a 12-h light/dark cycle with a standard mice chow diet and distilled water *ad libitum* for 7 days. Then, the mice were randomly divided into five groups (*n* = 6 per group): a normal control group (NC), a 3.5% DSS group (DSS, molecular weight: 36,000–50,000; MP Biomedicals), a 3.5% DSS + *B. longum* DD98 group (DD98), a 3.5% DSS + Se-*B. longum* DD98 (SeDD98), and a sulfasalazine group (SASP). Mice in the DSS group and treatment groups were treated with normal drinking water before DSS administration (day −14 to 0) followed by 3.5% DSS water for 7 days to induce colitis (day 0–7). Then, 3.5% DSS water was replaced by normal drinking water from day 7 to 9. In comparison, mice in the NC group were treated with normal drinking water throughout the whole experiment. For treatment, mice in the DD98 and SeDD98 groups were separately administered *B. longum* DD98 (1 × 10^10^ CFU/kg) and Se-*B. longum* DD98 (0.1 mg Se/kg, 1 × 10^10^ CFU/kg) once daily by oral gavage for 21 days (day −14 to day 7), while sulfasalazine was orally administered to mice at the dose of 300 mg/kg at the same time in the SASP group. By contrast, the NC group and DSS group were supplied with the same amount of normal saline. Besides, the body weight, stool consistency, and rectal bleeding were recorded daily from day 0 to day 9. At the end of the experiment (day 9), all mice were humanely euthanized. After that, the weight of the spleen and the length of the colon were measured. Feces and serum were stored at −80°C until analysis. Colon segments were either fixed with 4% paraformaldehyde or stored at −80°C for further procedures.

### Disease activity index assessment

The severity of colitis was measured using DAI assessment, a scoring system that includes three parts: body weight loss (0–4), degree of intestinal bleeding (0–4), and stool consistency (0–4) (Wirtz et al., 2017). For observation of disease progression, the DAI scoring was performed daily from day 0 to day 9 of the experiment.

### Histological analysis

Colon tissue was fixed with 4% paraformaldehyde solution and dehydrated by ethanol with different concentration gradients. Fixed tissue was embedded in paraffin and sections with a thickness of 4 μm were cut and stained with hematoxylin and eosin (H&E) followed by microscopic observations (Olympus Corporation, Tokyo, Japan). The colon damages were scored according to the criteria shown in Supplementary Table 1 as previously described (Stillie and Stadnyk, 2009). Scores were calculated by summing the score for four parameters (extent of inflammation, crypt damage, ulceration, and edema).

### Total RNA extraction and real-time quantitative PCR

Total RNA was extracted from tissues using TRIzol following manufacturer instructions. The concentrations of total RNA and the ratios of 260/280 and 260/230 were measured using Nanodrop (Thermo Fisher Scientific, MA, United States) to evaluate the quality of extracted RNA. The primers used to amplify the target genes were listed in Supplementary Table 2. The qPCR assays were performed using a StepOnePlus™ Real-Time PCR System (Thermo Fisher Scientific, MA, United States). The results were expressed as a relative value after normalization to the mRNA of GAPDH.

### Measurement of oxidative stress parameters in serum and colon

Malondialdehyde (MDA) concentration, superoxide dismutase (SOD), and Catalase (CAT) activity in serum were quantified using commercial kits (Nanjing Jiancheng Bioengineering Institute, Nanjing, China). The level of Glutathione reductase (GR) activity in colon tissue samples was measured by a commercial kit (Beyotime Biotechnology, Shanghai, China). All operations followed the kit’s instructions.

### Western blot analysis

Tissues were lysed in RIPA buffer with the addition of protease and phosphatase inhibitor cocktail (200 mM AEBSF, 30 μM Aprotinin, 13 mM Bestatin, 1.4 mM E64, 1 mM Leupeptin in DMSO, and 0.1 M EDTA). The lysate was centrifuged at 12,000 g at 4°C for 30 min and the protein concentration was measured using a BCA protein quantitative reagent kit (Beyotime, Shanghai, China). An equal amount of protein from each sample was separated by 8% SDS-PAGE and transferred to a PVDF membrane (Millipore, MA, United States). After blocking the membrane with 5% skimmed milk in tris buffered saline with tween-20 (TBST), the membrane was incubated with primary antibody against occludin, ZO-1, and TLR4 (Proteintech Group Inc., IL, United States) overnight at 4°C. GAPDH was used as a control. The membranes were then washed three times with TBST before being probed with the horseradish peroxidase-conjugated secondary antibodies. Signals were detected using a chemiluminescence system (ECL; Bio-rad, CA, United States; Tanon 4600, China) and the intensities were quantified using the ImageJ software.

### 16S gut microbiota profiling

Samples of mice colon contents of all groups were taken out and three mice in DSS and five mice in other groups were sequenced. Total bacterial DNA was extracted from fecal samples using a QIAamp Fast DNA Stool Mini Kit (Qiagen, Hilden, Germany) following the manufacturer’s procedures. The V3-V4 hypervariable region of the bacterial 16S rRNA gene was amplified for species classification by PCR using the specific primer. Illumina Miseq PE300 was used for sequencing after purification and library construction. Then, high-throughput sequencing was used to analyze the sample data. Alpha and beta-diversity were calculated to reflect the species richness in each group as well as to evaluate the similarity or difference of bacterial composition among groups. Linear discriminant analysis Effect Size (LEfSe) was analyzed to assay the species differences.

### Statistical analysis

All data were presented as the mean ± standard error of the mean (SEM). Statistical analysis was performed by the SPSS 26.0 statistical software. The difference between groups was analyzed by the one-way analysis of variance (ANOVA) or Kruskal–Wallis tests, and *p* < 0.05 was considered to be statistically significant.

## Results

### Effect of selenium-enriched *Bifidobacterium longum* DD98 on symptoms of the dextran sulfate sodium-induced colitis

**FIGURE 2:**
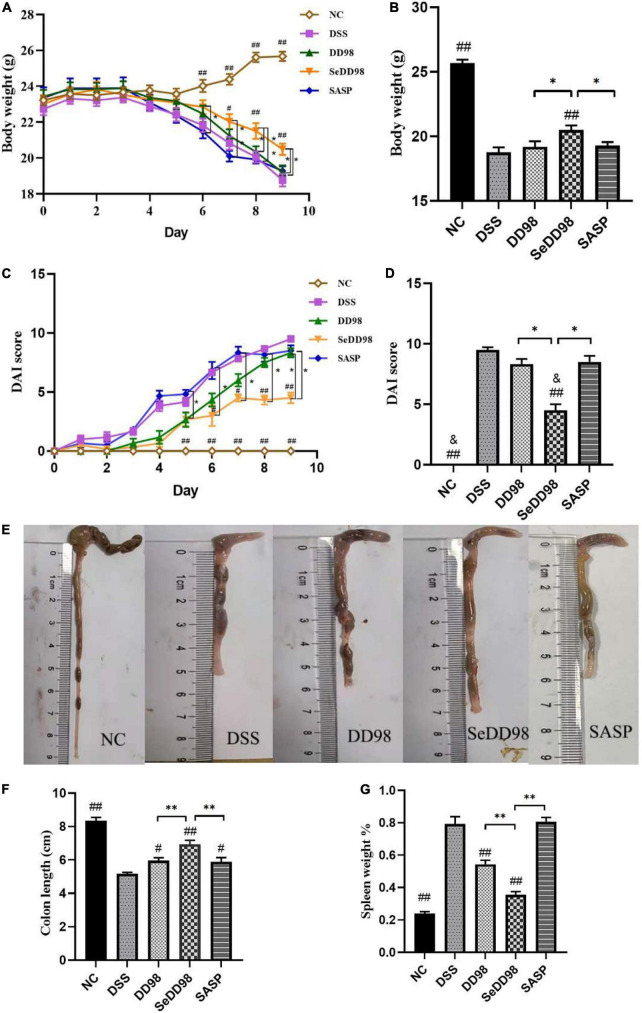
Effects of Se-*B. longum* DD98 on colitis symptoms in mice (*n* = 6). **(A)** Body weight change. **(B)** Body weight among the groups at day 9. **(C)** The disease activity index (DAI) scores calculated with time. **(D)** The DAI scores among the groups at day 9. **(E)** Representative images of colon tissues. **(F)** Data of the colon length. **(G)** The ratio of spleen weight to body weight. Data are shown as the mean ± SEM. *p < 0.05, ^**^p < 0.01. ^#^*p* < 0.05 compared to DSS group. ^##^*p* < 0.01 compared to DSS group. ^&^*p* > 0.05 compared to NC group. Body weight, colon length, and the percentage of spleen weight were analyzed by one-way ANOVA. DAI score was analyzed by Kruskal–Wallis tests.

As reported previously, body weight, DAI, and colon length reflected the severity of inflammation in DSS-induced colitis. The average body weight of each group during this experiment can be seen in Supplementary Table 3. The body weight of each mouse was recorded daily after 3.5% DSS water administration. Compared to the initial weight on day 0, the body weight of mice in another four groups except the NC group was consistently reduced since day 5 (Figure 2A). Mice in the DSS model group exhibited significant weight loss relative to untreated controls. However, Se-*B. longum* DD98 treatment significantly alleviated weight loss (Figure 2B). Similarly, DSS treatment remarkably increased the DAI scores and administration of Se-*B. longum* DD98 markedly decreased the DAI scores compared to the DSS, DD98, and SASP groups (Figures 2C,D). Following 7 days of DSS exposure, the colons of the mice were significantly shortened. Treatments of *B. longum* DD98, Se-*B. longum* DD98, and sulfasalazine significantly increased the length of colon and Se-*B. longum* DD98 exhibited a higher distinct effect (*p* < 0.05) (Figures 2E,F).

Of note are the spleens from mice that received DSS for 7 days which were significantly enlarged compared to untreated control. Mice from the DD98 displayed mild splenomegaly and the mice in SASP group suffered severe splenomegaly comparable to the DSS group. While, spleen weight of mice in SeDD98 group was significantly lighter than the other treatment (Figure 2G). Taken together, these data suggested that Se-*B. longum* DD98 treatment significantly improved UC-related parameters.

### Selenium-enriched *Bifidobacterium longum* DD98 alleviated histopathological damage in colitis mice

**FIGURE 3:**
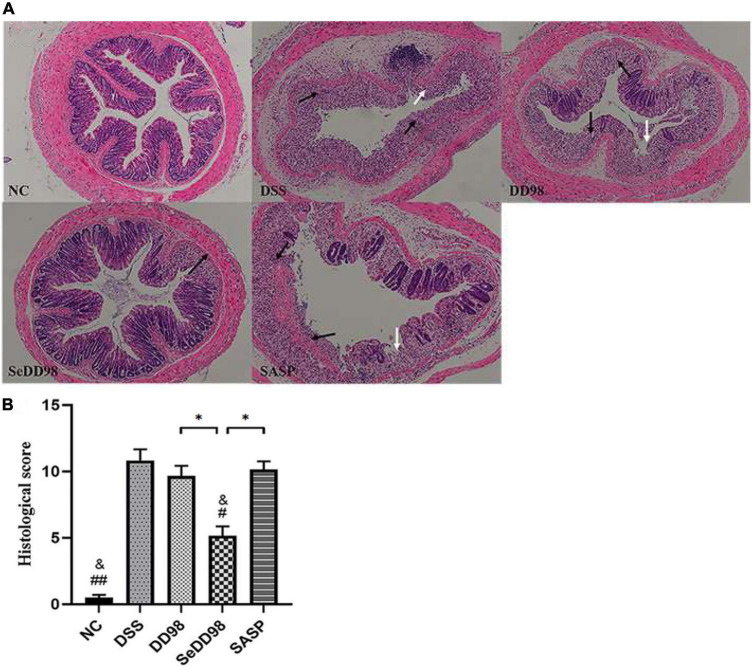
**(A)** Representative photomicrographs of H&E-stained sections of colons. Black arrow, area of strong transmural inflammation with loss of crypt structure and depletion of goblet cells; white arrow, ulceration. **(B)** Histopathology scores of colon tissues (*n* = 6). Data are shown as the mean ± SEM. *p < 0.05. ^#^*p* < 0.05 compared to DSS group. ^##^*p* < 0.01 compared to DSS group. ^&^*p* > 0.05 compared to NC group. Histological score was analyzed by Kruskal–Wallis tests.

To better evaluate the effect of Se-*B. longum* DD98 in reducing disease severity in DSS-colitis mice, histological examination of colon sections was also conducted. As shown in Figure 3A, the colon section of the DSS group exhibited extensive colonic damages, including neutrophilic infiltrates, ulceration, crypt abscess, and strong transmural inflammation with loss of crypt structure and depletion of goblet cells, while none of these pathological features were found in the NC group. Compared with the DSS group, the treatments of *B. longum* DD98, Se-*B. longum* DD98, and sulfasalazine could improve pathological injury to varying degrees. Furthermore, administration of *B. longum* DD98 and sulfasalazine induced less neutrophil infiltration and ulceration, partially preserved crypt structure and goblet cells in colon sections, and decreased scores in histological analysis although no significant difference. However, Se-*B. longum* DD98 treatment significantly improved these pathological damages and reduced histological scores (*p* < 0.05) (Figure 3B). Therefore, Se-*B. longum* DD98 could alleviate colon damage in mice with colitis, which was more effective than *B. longum* DD98 and sulfasalazine.

### Effects of selenium-enriched *Bifidobacterium longum* DD98 on the inflammation-related gene expression

**FIGURE 4:**
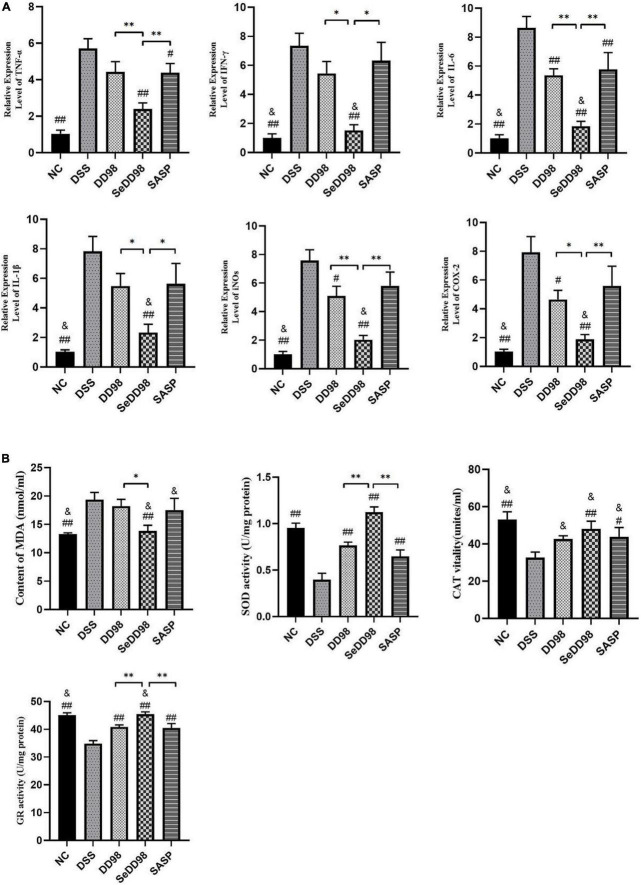
Effects of Se-*B. longum* DD98 on the relative expression levels of inflammation in the colon (*n* = 6) **(A)** and oxidative stress parameters in serum and colon (*n* = 6) **(B)**. Data are shown as the mean ± SEM. *p < 0.05, ^**^p < 0.01. ^#^*p* < 0.05 compared to DSS group. ^##^*p* < 0.01 compared to DSS group. ^&^*p* > 0.05 compared to NC group. TNF-α, IL-1β, and the activity of SOD, CAT, and GR were analyzed by one-way ANOVA. IFN-γ, IL-6, iNOs, COX-2, and MDA were analyzed by Kruskal–Wallis tests.

The effects of Se-*B. longum* DD98 on the expression of genes involved in inflammation in the colon is measured by RT-qPCR and shown in Figure 4A. The results showed that the expression of TNF-α, IFN-γ, IL-6, IL-1β, inducible nitric oxide synthases (iNOS), and cyclooxygenase-2 (COX-2) were significantly increased upon DSS administration, which indicated the severe inflammation of colon tissues in DSS mice. *B. longum* DD98 and sulfasalazine exerted different effects on these parameters compared to colitis mice in this study. For example, *B. longum* DD98 decreased the expression of IL-6, iNOS, and COX-2 in the colon significantly, while, sulfasalazine only exhibited a dramatically protective role in TNF-α and IL-6. Administration of Se-*B. longum* DD98 significantly reduced these six inflammatory genes expression and showed a stronger anti-inflammation action compared to *B. longum* DD98 and sulfasalazine. Something else we need to notice is that Se-*B. longum* DD98 reverted the gut inflammation to normal status, which was inferred from the expression of IFN-γ, IL-6, IL-1β, iNOs, and COX-2 in the SeDD98 group, which was in accordance with that in the NC group (*p* > 0.05). In general, Se-*B. longum* DD98 could regulate colon inflammation to the levels of the NC group and show a higher ability in decreasing inflammation parameters than *B. longum* DD98 and sulfasalazine.

### Effects of selenium-enriched *Bifidobacterium longum* DD98 on oxidative stress in serum and colon

Oxidative stress plays an important role in the progression of colitis. To better understand the mechanism of the protection of Se-*B. longum* DD98 against oxidative stress, we determined the content of MDA as well as the activities of SOD and CAT in serum and the activity of GR in colon (Figure 4B). The results showed that the content of MDA, which reflects the degree of lipoperoxidation in the host, was significantly increased in the DSS group, indicating the elevated oxidative stress induced by colitis. Among all treatments, Se-*B. longum* DD98 exerted greater influence in bringing down the aberrant level of MDA to the same level as the NC group. What’s more, the activities of SOD, CAT, and GR, mirroring the ability of scavenging oxygen-free radicals and resisting oxidative stress, were significantly decreased upon DSS administration. However, their activities could be restored by Se-*B. longum* DD98 treatment. In brief, Se-*B. longum* DD98 exerted significantly greater ability in maintaining the redox balance than *B. longum* DD98 and sulfasalazine.

### The impact of selenium-enriched *Bifidobacterium longum* DD98 on intestinal barrier and TLR4 in mice

**FIGURE 5:**
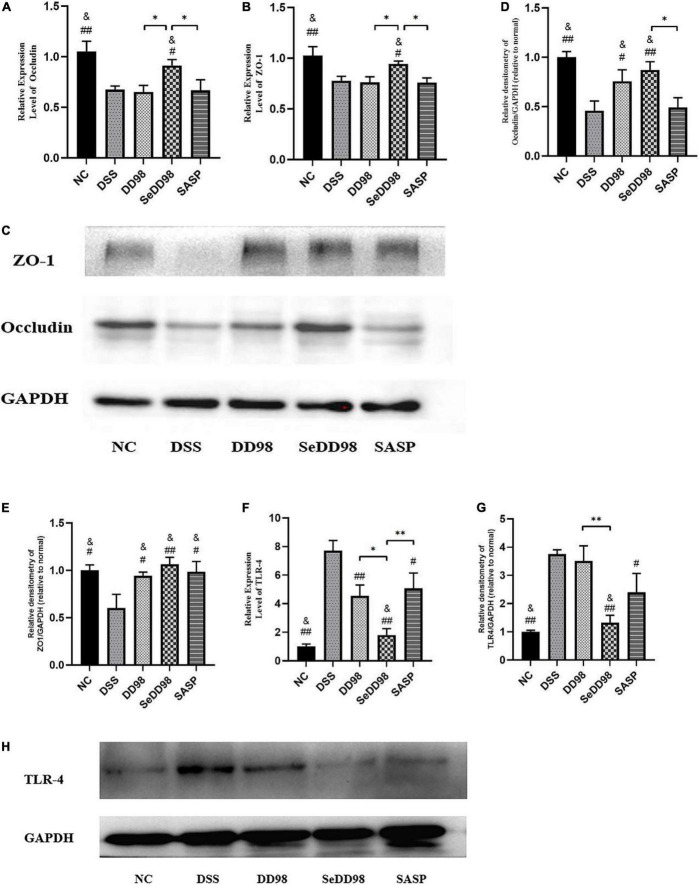
The impact of Se-*B. longum* DD98 on intestinal barrier function in DSS-treated mice. **(A,B)** Occludin and ZO-1 mRNA expression levels were assessed *via* RT-qPCR (*n* = 6). **(C–E)** Occludin and ZO-1 levels in colon tissues were assessed *via* western blotting (*n* = 3). **(F)** TLR4 mRNA expression level was assessed *via* RT-qPCR (*n* = 6). **(G,H)** TLR4 level in colon tissues was assessed *via* western blotting (*n* = 3). Data are shown as the mean ± SEM. *p < 0.05, ^**^p < 0.01. ^#^*p* < 0.05 compared to DSS group. ^##^*p* < 0.01 compared to DSS group. ^&^*p* > 0.05 compared to the NC group by one-way ANOVA.

The impact of Se-*B. longum* DD98 on intestinal barrier function was further investigated. To evaluate the relationship between Se-*B. longum* DD98 treatment and tight junction integrity in the intestines, we adopted RT-qPCR and western blotting to assess the expression of genes and protein levels of occludin and ZO-1. As shown in Figure 5, both gene and protein expressions of occludin (Figures 5A,C,D) and ZO-1 (Figures 5B,C,E) in the DSS group were significantly decreased compared with that in the NC group. However, *B*. *longum* DD98, Se-*B. longum* DD98, and sulfasalazine treatments could increase their expression, especially for Se-*B. longum* DD98 (Figures 5C–E). These results may explain the reasons for Se-*B. longum* DD98 with higher activity.

Increasing studies suggested that activation of toll-like receptors (TLRs) leads to increased expression of inflammatory cytokines and chemokines, contributing to the inflammatory response in colitis (Kordjazy et al., 2018). We found that both mRNA and protein expression levels of TLR4 were elevated in DSS-induced colitis mice (Figures 5F,G), corresponding to the results of increased inflammation caused by DSS. Se-*B. longum* DD98 inhibited TLR4 expression to the normal level, showing a better property in decreasing inflammatory reaction compared to *B. longum* DD98 and sulfasalazine.

### The effect of selenium-enriched *Bifidobacterium longum* DD98 on the gut microbiota in colitis mice

**FIGURE 6:**
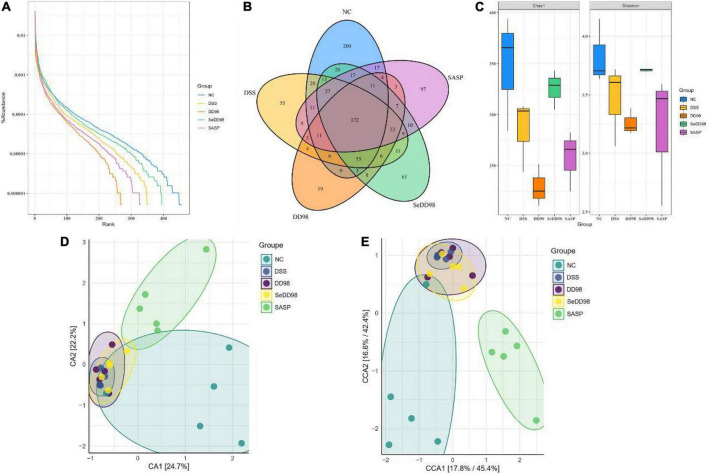
The effect of Se-*B. longum* DD98 on the intestinal microbiota (*n* = 3–5). **(A)** Rank-abundance curve. **(B)** Venn diagram. Totally 172 gut microbial species were shared by the five groups of mice, and there were 200, 55, 19, 61, and 97 distinct microbes (ASVs) in the NC, DSS, DD98, SeDD98, and SASP groups, respectively. **(C)** Chao and Shannon index. **(D,E)** CA and CCA analysis.

To characterize the changes in the gut microbiota composition, the 16S rDNA high-throughput sequencing approach was employed. Rank-abundance curve, reflecting both abundance and evenness of specie, is a way of analyzing diversity in intestinal flora analysis. As shown in Figure 6A, the curve revealed that Se-*B. longum* DD98 treatment showed an advantage in increasing the abundance and evenness of gut microbiota in colitis mice. According to the Venn diagram shown in Figure 6B, 172 gut microbial species were shared by the five groups of mice, and there were 200, 55, 19, 61, and 97 distinct microbes (ASVs) in the NC, DSS, DD98, SeDD98, and SASP groups, respectively. Analyses of α-diversity demonstrated that mice in the SeDD98 group exhibited increased intestinal flora richness and diversity compared with DSS, DD98, and SASP groups. However, it is worth noting that the diversity of microbiota in the DD98 group is the lowest among these groups (Figure 6C). β-diversity represents the similarity of microbial composition among different groups, and the β-diversity analysis showed that the similarity of microbial composition between the NC group and the SASP group was good. While, there is a great difference in microbial composition between the NC group and the DSS group and the difference was reduced after Se-*B. longum* DD98 administration (Figures 6D,E).

**FIGURE 7:**
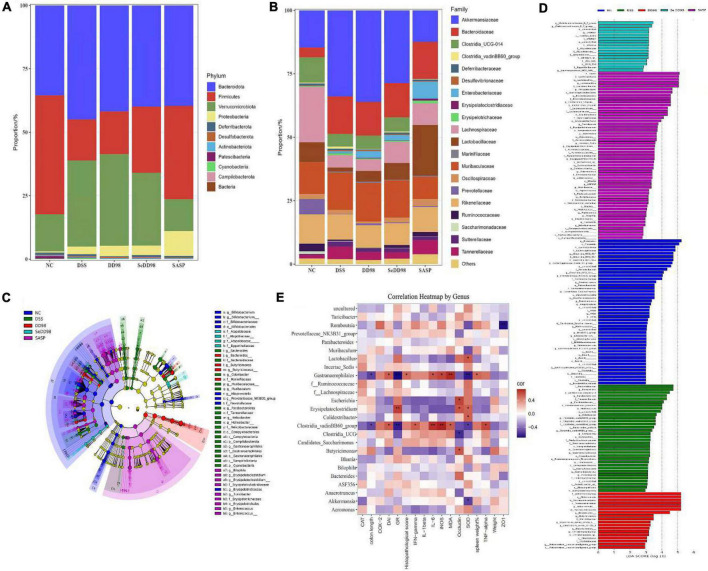
Relative abundance of taxa at the phylum **(A)** and family **(B)** levels; Cladogram of LEfSe analysis **(C)** and Histogram of the LDA scores **(D)**; Correlation between gut microbiota and colitis parameters by Spearman correlation analysis. *Spearman’s correlation is significant at a level of 0.05; ^**^Spearman’s correlation is significant at a level of 0.01 **(E)** (*n* = 3–5).

As shown in Figure 7A, the phylum of *Bacteroidetes* decreased and the phylum of *Firmicutes* increased after Se-*B*. *longum* DD98 administration compared with the DSS group. At a family level (Figure 7B), an increase in *Akkermansiaceae*, *Bacteroidaceae*, and *Muribaculaceae* and a decrease in *Clostridia-UCG014*, *Lachnospiraceae*, *Lactobacillaceae*, and *Prevotellaceae* in DSS group was observed, and this dysbiosis was altered after Se-*B*. *longum* DD98 treatment. The LEfSe analysis was also conducted to identify groups of bacteria that differed significantly among groups (Figures 7C,D). *Bacteroidaceae* in the DSS group and *Christensenellaceae* and *Lachnospiraceae* in the SeDD98 group were specifically abundant. Spearman’s correlation analysis was employed to investigate the correlation of gut microbiota at a genus level with colitis parameters. It was found that 10 genera were at least negatively or positively correlated with one parameter of IBD, including DAI score, transcriptional expression of inflammatory factors, oxidative stress indicators, colon length, the percentage of spleen weight, histopathological score, and intestinal integrity-related genes in intestinal tissue (Figure 7E). For instance, *Lactobacillus*, *Erysipelatoclostridium*, and *Colidextribacter* was significantly positively correlated with SOD activity. *Romboutsia* was negatively correlated with ZO1 expression. *Gastranaerophilales* have a very significant positive correlation with MDA, iNOS, IL-6, histopathological score, and DAI score and a significant negative correlation with colon length, GR, and SOD activity.

## Discussion

**FIGURE 8:**
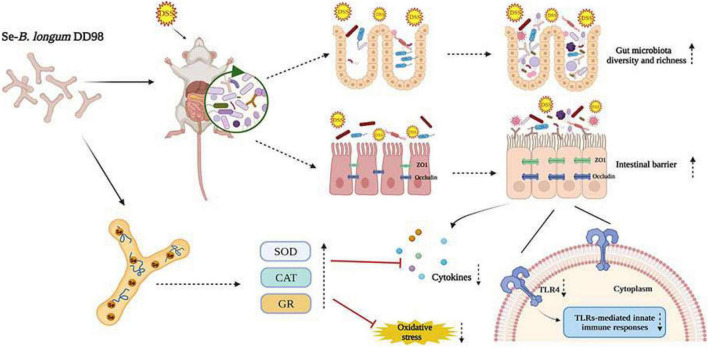
Proposed mechanisms of Se-*B*. *longum* DD98 in the inhibition of DSS-induced colitis in mice.

Ulcerative colitis is characterized by a non-specific chronic intestinal inflammation, generally occurring in the distal colon and rectum. The pathogenesis is not entirely understood and is usually caused by multi-factors including genetic predisposition, intestinal flora dysbiosis, epithelial barrier defects, immune susceptibility, and environmental elements (Ungaro et al., 2017). Therapeutic drugs for UC are not effective enough and associate with many adverse reactions. Probiotics such as *Lactobacillus* and *Bifidobacterium* are safe and proven effective in the treatment of UC by mediating microbiota dysbiosis, enhancing intestinal integrity and generating functional metabolites (Sanders et al., 2019). Additionally, as an essential trace element, selenium, especially the organic selenium, is famous for its antioxidative properties and effect on regulating immune systems, thus playing a positive role in UC treatment (Ala and Kheyri, 2021). In this report, we found that Se-*B. longum* DD98, which combines the properties of probiotics and organic selenium, was able to efficiently alleviate symptoms caused by DSS-induced colitis. As Figure 8 showed, Se-*B. longum* DD98 functioned as the gut microbiota regulator, which was proven to increase the diversity and richness of gut microbiota in DSS-treated mice. Se-*B. longum* DD98 could also enhance tight junction protein expression, such as ZO-1 and Occludin, thus improving the intestinal barrier integrity in colitis mice. Besides, we found that Se-*B. longum* DD98 significantly inhibited the TLR4 expression in colon tissues of UC mice, which was deemed to be a key factor in decreasing pro-inflammatory cytokines. Furthermore, the selenoproteins, that Se-*B. longum* DD98 contains, were famous for its anti-oxidation effect, which was certified by enhanced activity of SOD, CAT, and GR, and decreased MDA content induced by Se-*B. longum* DD98 in this text.

It is reported that DSS colitis caused severe symptoms characterized by weight loss, bloody diarrhea, and abdominal pain that resemble typical features in UC patients (Wirtz et al., 2017). Of note is that although sulfasalazine is one of the first-line drugs in IBD treatment, its effectiveness is just modest and approximately 20% of UC patients are intolerant to this treatment due to its adverse effects (Ye and van Langenberg, 2015; Cai et al., 2021). Besides, lifelong medication was recommended for relapse prevention in mild-to-moderate UC patients (Ko et al., 2019). It also has been reported that the application of sulfasalazine exerted protective effects in DSS-induced colitis mice, whereas there was little significance (Shin et al., 2017). In this study, sulfasalazine showed a modest therapeutic effect on body weight and DAI. Whereas, treatment of sulfasalazine significantly improved the colon length, the activity of SOD, CAT, and GR, and the expression of ZO-1 in colitis mice. What’s more, sulfasalazine significantly decreased the expression of pro-inflammatory cytokines, such as TNF-α and IL-6, as well as TLR4 activation in colon tissue compared with DSS-treated mice, indicating the effective but not satisfactory enough effect of sulfasalazine in UC treatment, which was consistent with the utilization status of sulfasalazine in IBD patients. Our findings were in the accordance with related reports, which called for a more efficient treatment both in colitis mice and UC patients. Herein, we demonstrated that Se-*B. longum* DD98 treatment significantly reduced weight loss, colon shortening, splenomegaly, and improved DAI scores and histological scores induced by DSS colitis. Se-*B. longum* DD98 showed a greater advantage in treatment compared with *B. longum* DD98 and sulfasalazine.

It has been pointed out that proinflammatory cytokines and oxidative stress play an important role in the initiation and evolution of IBD (Strober and Fuss, 2011; Wang et al., 2016). And the supplement of selenium or probiotics could alleviate inflammatory cytokines and preserve the microstructure of colon (Kaur et al., 2018; Bian et al., 2019). TNF-α is the major meditator of inflammation and specific blockade of TNF-α with drugs such as infliximab and etanercept were demonstrated to have great efficacy in the treatment of IBD patients (Yao et al., 2019). IFN-γ, IL-1β, and IL-6 also play a crucial role in the development of colitis. Peng et al. reported that anthocyanins from the fruits of *Lycium ruthenicum* Murray decreased mRNA of TNF-α, IL-6, IFN-γ, and IL-1β in DSS-induced colitis in mice (Peng et al., 2019). Our study was in line with this research and demonstrated that the inhibitory effect of Se-*B. longum* DD98 on mRNA expression of proinflammatory cytokines was better than *B. longum* DD98 and sulfasalazine in colitis mice. NO is also an important pro-inflammatory initiator generated by iNOS in damaging tissue cells and leading to inflammation. A reduction of expression of iNOS is beneficial to alleviate oxidative stress and inflammation. A recent study demonstrated that enhanced COX-2 expression was observed in colonic epithelial tissues of UC patients and selenium decreased COX-2 expression by activating AMP-activated protein kinase (Hwang et al., 2006; Lin et al., 2018). Therefore, the change of COX-2, which was decreased to a normal level after Se-*B. longum* DD98 treatment, was detected by expression at the mRNA level in the present study. Besides, UC is complicated with a large amount of generation of reactive oxygen species (ROS), leading to lipid peroxidation and MDA production. So, MDA reflects the degree of lipid peroxidation and cell injury. The endogenous antioxidant system such as SOD, CAT, and GR can reduce oxidative stress and ROS production directly or indirectly. It is widely recognized that selenoproteins exert an antioxidant effect by participating in the synthesis of antioxidant enzymes (Ala and Kheyri, 2021). In the present study, the results showed that the content of MDA was significantly increased after DSS administration. At the same time, SOD, CAT, and GR activities were significantly inhibited in the DSS group. Administration of Se-*B. longum* DD98 exerted greater ability in regulating the aberrant level of the aforementioned parameters to the same level as the NC group, indicating a better inhibition effect on oxidative stress than probiotic *B. longum* DD98 and sulfasalazine.

Furthermore, IBD is complicated with a reduced intestinal barrier and an impaired intestinal barrier precedes symptoms for years, indicating that an impaired intestinal barrier contributed to the progression of this disease (Mehandru and Colombel, 2021). As a vital component of the intestinal barrier, tight junction (TJ) proteins play crucial roles in maintaining barrier integrity. The abnormal function of colonic TJ proteins, including the downregulation of ZO-1 and occludin, increases colon permeability and leads to the destruction of the intestinal barrier (Zeisel et al., 2019). *In vitro* and *in vivo* studies showed that supplementation of probiotics formed the microscopic scaffolds of the intestinal barrier, and enhanced intestinal barrier integrity and expression of tight junction proteins (Resta–Lenert and Barrett, 2006; Rose et al., 2021). Besides, Zhai et al. (2018b) reported that dietary selenium supplementation promoted the mRNA expression levels of TJ proteins in normal mice, which was confirmed by FMT to DSS-treated mice. In this study, DSS induced a dramatic reduction on the expression of occludin and ZO-1 in colon tissues, while Se-*B*. *longum* DD98 treatment significantly increased mRNA expression of TJ protein levels, which was ascertained by western-blotting results.

In DSS colitis mice, DSS exerted toxicity to the intestinal barrier and caused epithelial injury in the colon, giving opportunistic pathogens and luminal antigens a chance to pass through the impaired barrier and induce a rapid and profound inflammatory immune response (Wirtz et al., 2017). Therefore, TLR4, a receptor expressed in intestinal epithelial cells, recognized pathogen-associated molecular patterns (PAMPs) such as flagellin and lipopolysaccharide (LPS) and becomes activated, which leads to the over-generation of inflammatory cytokines and IBD (Kordjazy et al., 2018). Besides, the activation of TLR4 is known to induce NF-κB to transfer from cytoplasm to nucleus through MyD88-dependent or MyD88-independent pathways, thus initiating pro-inflammatory gene transcription and aggravating inflammation severity. It was also reported that TLR4 activation both had advantages and disadvantages. On one hand, minor activation of TLR4 is essential for immune homeostasis. While, over-expression of TLR4 can lead to the induction of host inflammatory responses, such as IBD (Luo et al., 2020). Therefore, decreasing the over-expression of TLR4 is an important target for the treatment of IBD. In the present study, the mRNA and protein levels of TLR4 were elevated in the intestines after DSS treatment. All interventions reversed the increased TLR4 level to a different degree, while mice in the SeDD98 group possessed the significantly lowest TLR4 level in the colon. Probiotics or selenium supplements have been reported to decrease the level of TLR4 expression (Bian et al., 2019; Chen et al., 2020), which is in line with our results that Se-*B. longum* DD98 exhibited a priority in decreasing the activation of TLR4 and related proinflammatory cytokines compared with probiotic *B. longum* DD98 and sulfasalazine.

Recent evidence showed that the pathogenesis of IBD was complicated by gut microbiota dysbiosis, an undesirable change in the composition of gut microbiota, which includes a reduction in the abundance of *Firmicutes* as well as increases in the abundance of *Proteobacteria* and *Bacteroidetes* (Ni et al., 2017; Nishida et al., 2018). Probiotics and selenium supplements are widely used to regulate intestinal flora in experimental colitis models (Bian et al., 2019; Ala and Kheyri, 2021). Furthermore, the intestinal microbiome is also associated with the integrity of the intestinal barrier and stable immune function. Herein, the composition of intestinal flora in mice among different treatment groups was investigated. We found that the community of composition of the microbiota from the DSS group is significantly different from the NC group. The Chao and Shannon index are important indicators of community richness and diversity. The results showed that after Se-*B. longum* DD98 treatment, the richness and diversity of gut microbiota were elevated. At the phylum level, the ratio of *Firmicutes* to *Bacteroidetes*, an index inversely related to the severity of IBD symptoms (Sartor and Wu, 2017), was increased after Se-*B. longum* DD98 treatment. At the family level, the administration of Se-*B. longum* DD98 altered the flora composition in DSS-treated mice, including a decrease in *Akkermansiaceae* and an increase in *Clostridia-UCG014*, *Lachnospiraceae*, *Lactobacillaceae*, and *Prevotellaceae*. A recent study pointed out that some enzymes secreted by *Akkermansiaceae* broke down the mucus in the lining of the large intestine, posing a risk to the intestinal barrier in the gut (Khan et al., 2020). *Clostridia-UCG014* is a probiotic associated with tryptophan metabolism. Its tryptophan catabolite indole-3-acetic acid (IAA) can activate the aryl hydrocarbon receptor (AhR), which was thought to exert a protective role in the gut barrier (Chengcheng et al., 2021). Besides, *Lactobacillaceae* has been widely recognized as a probiotic and used for the prevention of IBD for many years. *Prevotellaceae* was thought to participate in short chain fatty acid (SCFA) generation, which protected the intestinal barrier in IBD patients (Shen et al., 2017). Therefore, Se-*B. longum* DD98 may play a protective role in DSS-induced mice by upregulating the abundance of *Clostridia-UCG014*, *Lachnospiraceae*, *Lactobacillaceae*, and *Prevotellaceae*. It is interesting to note that the abundance and diversity of gut microbiota in the DD98 group were the lowest in these groups, which may be related to the relatively small sequencing samples. However, at the phylum level, the ratio of *Firmicutes* to *Bacteroidetes* was increased in the DD98 group compared with the DSS group. What’s more, supplement of *B. longum* DD98 upregulated the proportion of *Lactobacillaceae*, which could be connected with colitis improvement in mice. Additionally, LEFSe analysis was employed to identify the community with statistical differences between groups. Herein, we found that *Christensenellaceae* and *Lachnospiraceae* in the SeDD98 group were specifically abundant. *Lachnospiraceae* is a kind of SCFA-producing probiotic, which regulates the immune response and inflammatory response in the host. Furthermore, *Lachnospiraceae* was demonstrated to protect the colon barrier and is inversely related to diarrhea in patients (Guo et al., 2020). It is reported that *Christensenellaceae* is very important for host health and was a significantly negative correlation with inflammation, although the exact mechanism was unknown (Waters and Ley, 2019). What’s more, Spearman’s correlation analysis was employed to investigate the correlation of gut microbiota at a genus level with colitis parameters in our study. It is worth noting that *Gastranaerophilales* had a significant positive correlation with MDA, iNOS, IL-6, histopathological score, and DAI score and a significant negative correlation with colon length, GR, and SOD activity. However, little is known about this microorganism. There was also a study indicating that *Gastranaerophilales* is capable of converting glucose, mannose, starch, or glycogen into lactate, ethanol, and formate (Soo et al., 2014). Besides, one analysis of the correlation between gut microbiota and six common autoimmune diseases revealed that the family *Peptostreptococcaceae*, order *Gastranaerophilales*, and genus *Romboutsia* shared the most enriched taxa among five autoimmune diseases (Cao et al., 2021). Our research was in line with this and a relevant study should be the carrier to investigate the exact function of *Gastranaerophilales.* Although, Khattab et al. (2022) demonstrated the protective effect of selenium-enriched *Bifidobacterium longum* mutants on piroxicam-induced UC. However, there were many differences between these two studies. In all, Se-*B. longum* DD98 showed a better alleviation of symptoms, inflammation level, and oxidative stress, as well as a priority in protecting the intestinal barrier integrity and regulating the composition of gut microbiota in DSS-treated mice. We have compared the efficacy of Se-*B. longum* DD98 with Na_2_SeO_3_ (inorganic selenium), SeMet (organic selenium), or the combination of *B. longum* DD98 and SeMet during many experiments, and found that Se-*B. longum* DD98 exerted a superior effect than selenium or the combination in these different symptoms or diseases (Zhao et al., 2020a,b; Zhu et al., 2021). What’s more, in this study, Se-*B. longum* DD98 was treated as a whole, independent enhanced probiotic product. Meanwhile, the applications of probiotic or sulfasalazine are dominant in UC treatment, and selenium is seldom used in UC prevention alone due to its potential toxicity. So, we did not adopt selenium-treated group as a comparison in this study. Many studies have confirmed the protective effect of probiotics in UC *via* regulating gut microbiota dysbiosis. Lu et al. (2022) demonstrated that *Eurotium cristatum*, a probiotic, ameliorated DSS-Induced UC in mice by modulating the gut microbiota, and fecal bacteria transplantation (FMT) from probiotic-treated mice showed a similar protective effect in colitis mice, indicating that the microbiome changes result from probiotic supplement cause the alleviated inflammation in UC. What’s more, selenium was also determined to balance the gut microbiota avoiding health damage associated with dysbiosis (Ferreira et al., 2021). However, whether the restoration of gut microbiota after Se-*B. longum* DD98 administration is the cause or consequence of the alleviated inflammation in UC needs to be further confirmed. Germ-free mice and FMT should be adopted to investigate the role that gut microbiota plays in the alleviation of UC after Se-*B. longum* DD98 supplement. Further studies should be carried out to investigate the exact mechanism of how selenium strengthened the efficacy of probiotic *B. longum* DD98.

## Conclusion

In this study, we found that Se-*B. longum* DD98 effectively ameliorates DDS-induced colitis in mice. The effects were mainly due to alleviating symptoms caused by DSS, inhibiting the expression of the pro-inflammatory cytokines, decreasing the level of oxidative stress, promoting the expression of tight junction proteins, inhibiting the activation of TLR4, and regulating the gut microbiota. Furthermore, Se-*B. longum* DD98 presented greater protective effects than *B. longum* DD98 or sulfasalazine. In summary, Se-*B. longum* DD98 effectively attenuated DSS-induced colitis in mice, providing a potential therapeutic strategy for the treatment of IBD patients in the future.

## Data availability statement

The datasets for this study can be found in online repositories. The name of the repositories and accession number can be found below: NCBI BioProject accession no: PRJNA842916.

## Ethics statement

The animal study was reviewed and approved by Institutional Animal Care and Use Committee (IACUC) of Shanghai Jiao Tong University.

## Author contributions

YH: investigation, methodology, writing-original draft, and writing-review and editing. XJ: investigation and methodology. FG: investigation and software. TL: visualization and software. HZ: methodology. XH: investigation. YY: resources and project administration. SK: writing-review and editing. DC: supervision, project administration, and funding acquisition. All authors contributed to the article and approved the submitted version.
