# Supplementary material for: Selenium-enriched Bifidobacterium longum DD98 effectively ameliorates dextran sulfate sodium-induced ulcerative colitis in mice
Source: Front Microbiol. 2022 Aug 5;13:955112. doi: 10.3389/fmicb.2022.955112 (PMC9389208; doi:10.3389/fmicb.2022.955112)

Supplementary Material

# Supplementary Table S1. Histopathological Scores method

| Score | Inflamation | Crypt Damage | Ulceration | Edema |
| --- | --- | --- | --- | --- |
| 0 | No infiltrate | None | None | Absent |
| 1 | Occasional cell limited to submucosa | Some crypt damage, spaces between crypts | Small, focal ulcers | Present |
| 2 | Significant presence of inflammatory cells in submucosa, limited to focal areas | Large spaces between crypts, loss of goblet cells, some shorting of crypts | Frequent small ulcers |  |
| 3 | Infiltrate present in both submucosa and lamina propria, limited to focal areas | Large areas without crypts, surrounded by normal crypts | Large areas lacking surface epithelium |  |
| 4 | Large amount of infiltrate in submucosa, lamina propria and surrounding blood vessels, covering large areas of mucosa | No crypts |  |  |
| 5 | Transmural inflammation (mucosa to muscularis) |  |  |  |

**Supplementary Table S2. Special primers used for the RT-PCR analyses**

| Gene | Forward Sequence (5'-3') | Reverse Sequence (5'-3') |
| --- | --- | --- |
| TNF-α | AGGCACTCCCCCAAAAGAT | CAGTAGACAGAAGAGCGTGGTG |
| IFN-γ | TCAAGTGGCATAGATGTGGAAGAA | TGGCTCTGCAGGATTTTCATG |
| IL-6 | TAGTCCTTCCTACCCCAATTTCC | TTGGTCCTTAGCCACTCCTTC |
| IL-1β | AGCTTCAAATCTCGCAGCAG | TCTCCACAGCCACAATGAGT |
| iNOS | GGGCTGACCTGTTTCCTACT | GGAGGTTGAGACCCAATGGA |
| COX-2 | CCCATTAGCAGCCAGTTGTC | CAGGATGCAGTGCTGAGTTC |
| Occludin | TTCCTCTGACCTTGAGTGTGG | CTCTTGCCCTTTCCTGCTTT |
| ZO-1 | GCCGCTAAGAGCACAGCAA | GCCCTCCTTTTAACACATCAGA |
| TLR4 | GCTCTCAGCCATCCACAAAG | GAGTCGGGAAGAGGAAGAGG |
| β-actin | AGTGTGACGTTGACATCCGT | GCAGCTCAGTAACAGTCCGC |

**Supplementary Table S3. Average body weight in each group during the experiment.**

| Group | -14 | -7 | 0 | 1 | 2 | 3 | 4 | 5 | 6 | 7 | 8 | 9 |
| --- | --- | --- | --- | --- | --- | --- | --- | --- | --- | --- | --- | --- |
| NC | 21.51 | 22.12 | 23.22 | 23.57 | 23.50 | 23.67 | 23.77 | 23.57 | 24.02 | 24.39 | 25.61 | 25.67 |
| DSS | 21.61 | 22.08 | 22.73 | 23.31 | 23.21 | 23.38 | 22.88 | 22.42 | 21.82 | 20.82 | 20.05 | 18.76 |
| DD98 | 21.70 | 22.80 | 23.43 | 23.84 | 23.84 | 23.89 | 23.36 | 23.17 | 22.47 | 21.24 | 20.34 | 19.20 |
| SeDD98 | 21.56 | 22.42 | 23.01 | 23.55 | 23.83 | 23.53 | 23.26 | 23.08 | 22.84 | 22.03 | 21.50 | 20.49 |
| SASP | 21.88 | 22.47 | 23.35 | 23.89 | 23.89 | 23.90 | 23.09 | 22.36 | 21.48 | 20.10 | 19.91 | 19.28 |

**The following were supplementary figures for western blot:**

C57BL/6 mice Occludin: *left* NC DSS DD98 SeDD98 SASP *right*

C57BL/6 mice GAPDH: *left* NC DSS DD98 SeDD98 SASP *right* (Bio-rad, USA)


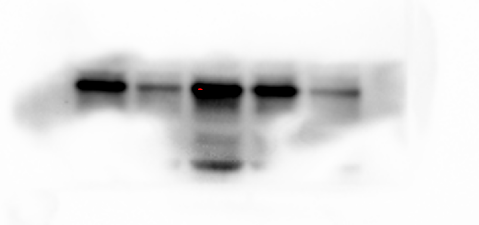

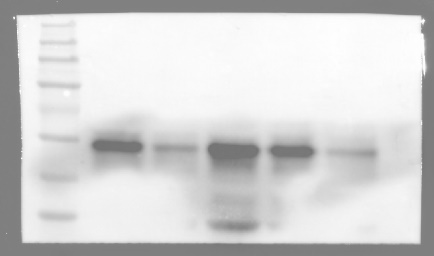

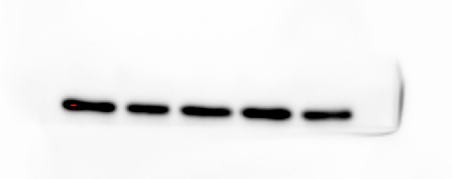

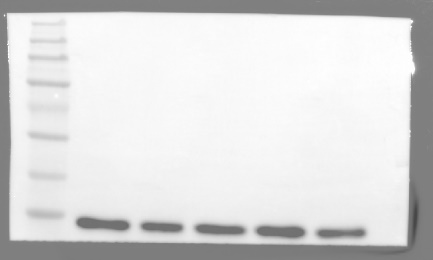

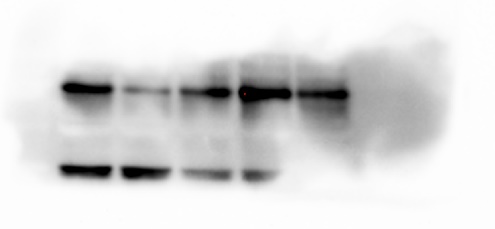

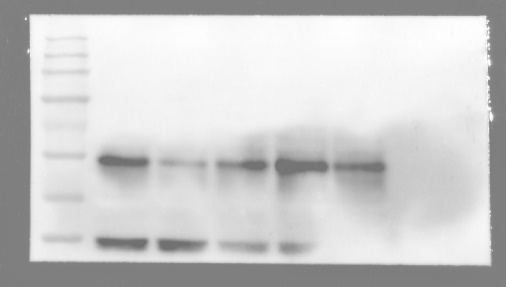

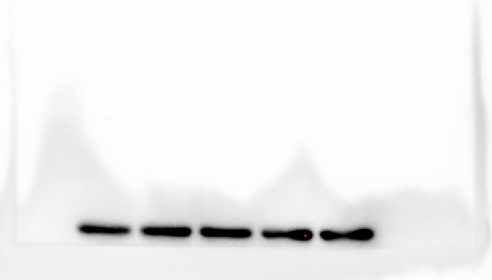

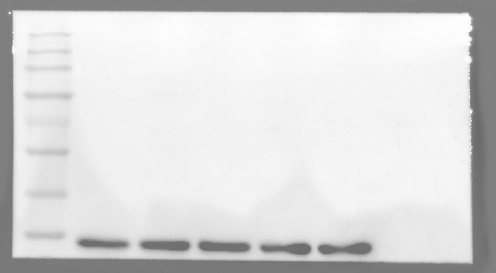

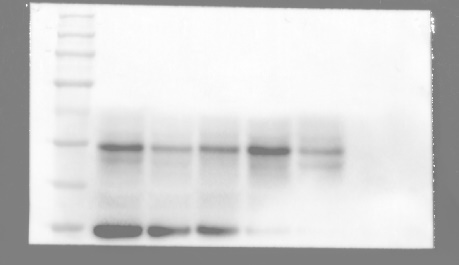

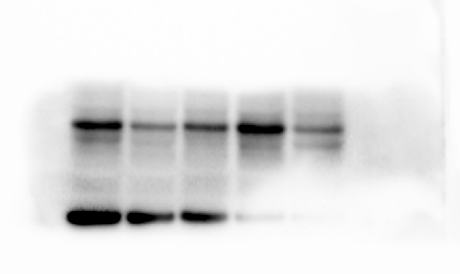

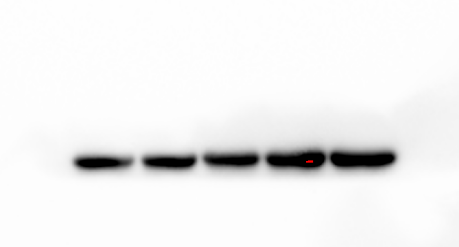

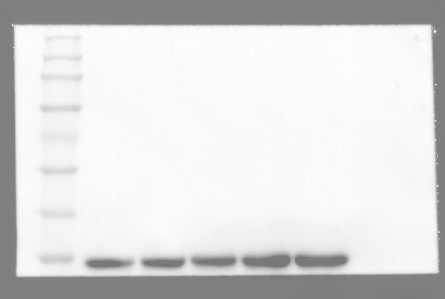


C57BL/6 mice ZO-1: *left* NC DSS DD98 SeDD98 SASP *right*

C57BL/6 mice GAPDH: *left* NC DSS DD98 SeDD98 SASP *right* (Tanon 4600, China)


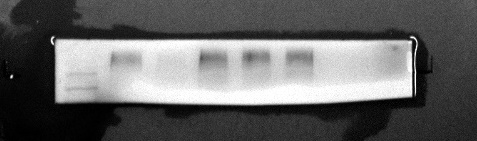

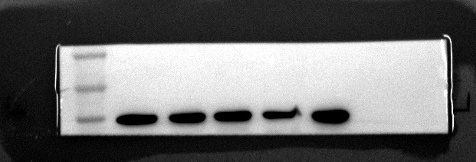

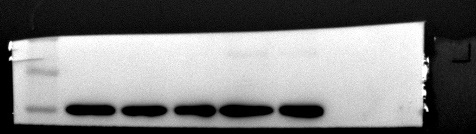

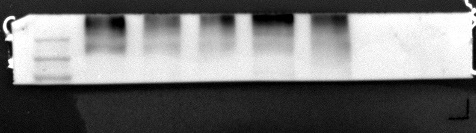

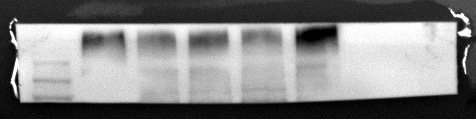

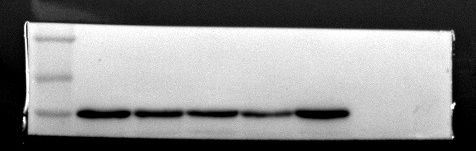


C57BL/6 mice TLR4: *left* NC DSS DD98 SeDD98 SASP *right*

C57BL/6 mice GAPDH: *left* NC DSS DD98 SeDD98 SASP *right* (Tanon 4600, China)


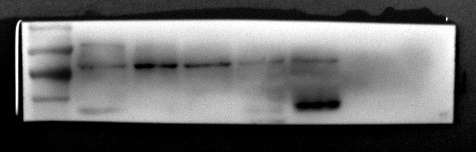

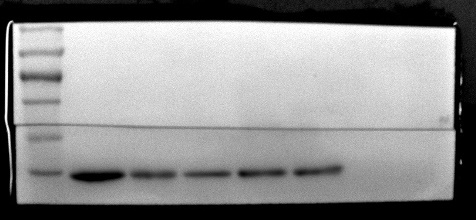

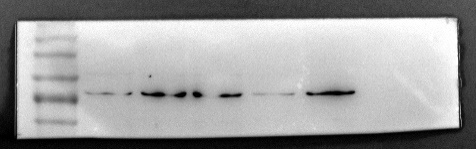

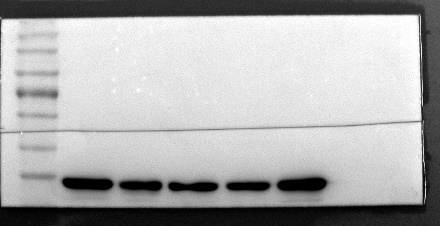

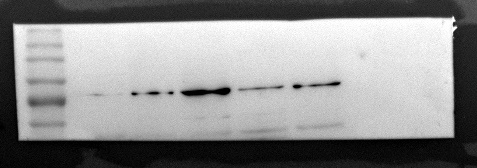

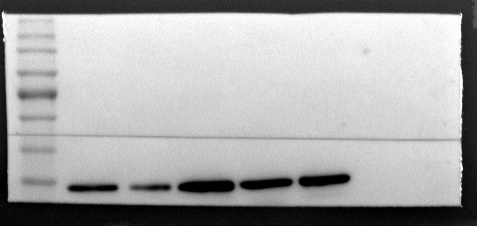

Supplement: Supplementary file 1 [file Table_1.docx]
